# The Critical Role of IL-10 in the Antineuroinflammatory and Antioxidative Effects of *Rheum tanguticum* on Activated Microglia

**DOI:** 10.1155/2018/1083596

**Published:** 2018-04-26

**Authors:** Jie Meng, Junjun Ni, Zhou Wu, Muzhou Jiang, Aiqin Zhu, Hong Qing, Hiroshi Nakanishi

**Affiliations:** ^1^Department of Aging Science and Pharmacology, Faculty of Dental Sciences, Kyushu University, Fukuoka 812-8582, Japan; ^2^OBT Research Center, Faculty of Dental Sciences, Kyushu University, Fukuoka 812-8582, Japan; ^3^Institution of Geriatric, Qinghai Provincial Hospital, Xining 810007, China; ^4^School of Life Science, Beijing Institute of Technology, Haidian District, Beijing 100081, China

## Abstract

*Rheum tanguticum* Maxim. ex Balf. (*Rt*), a traditional Tibetan medicine, is known to exert various bioactivities, including anti-inflammatory and antioxidative activities. The present study was conducted to investigate anti-inflammatory and antioxidative effects of *Rt* on activated microglia. *Rt* (10 *μ*g/ml) significantly inhibited the mean protein level of interleukin-1*β* (IL-1*β*) in the organotypic hippocampal slice cultures following treatment with chromogranin A (CGA, 10 nM) and pancreastatin (10 nM), endogenous microglial activators present in senile plaques. *Rt* also significantly inhibited the expression and production of inflammatory and oxidative molecules, including IL-1*β*, tumor necrosis factor-*α*, and nitric oxide, by cultured microglia after treatment with CGA. These effects of *Rt* are considered to be mediated by the secretion of interleukin-10 (IL-10) from microglia, because neutralizing antibodies against IL-10 significantly canceled these effects. To explore the causative components of *Rt* responsible for inducing the secretion of IL-10, the effects of seven components of *Rt* on the IL-10 expression in microglia were examined. Among them, aloe-emodin (10 *μ*M) and (+)-catechin (30 *μ*M) were able to induce the secretion of IL-10 from cultured microglia. Therefore, aloe-emodin and (+)-catechin are deemed responsible for the antineuroinflammatory and antioxidative effects of *Rt* through the secretion of IL-10 from microglia. Accordingly, *Rt* is considered potentially useful for the treatment of AD.

## 1. Introduction

There is increasing evidence that chronic neuroinflammation by activated microglia is closely associated with many neurological disorders, including Alzheimer's disease (AD). Furthermore, findings suggest that neuroinflammation mediated by activated microglia plays an essential role in the pathogenesis and progression of AD [1, 2]. Recently, it has been demonstrated that neuroinflammation is not a passive system activated by emerging senile plaques and neurofibrillary tangles but instead contributes as much to pathogenesis as do the plaques and tangles themselves [3]. More recently, two research groups have provided evidence that neuroinflammation is not a result of AD as much as a key driver of the disease [4, 5].

Medicinal plants can be considered an important resource for identifying anti-inflammatory agents, as they contain many kinds of natural polyphenols that exert anti-inflammatory and antioxidative activities. There is accumulating evidence indicating that medicinal plants and natural products including ginsenosides from *Panax ginseng*, curcumin from *Curucuma longaI*, and resveratrol, a natural polyphenol, have antineuroinflammatory and neuroprotective effects through inhibition of microglial activation [6]. Furthermore, we have recently reported that green propolis, a resinous substance by honeybees as a defense against intruders, has both antineuroinflammatory and neuroprotective effects [7, 8]. *Rheum tanguticum* (*Rt*), which is endemic to the eastern part of the Qinghai-Tibet Plateau in China, is a well-known traditional medicine with purgation, antibacterial, antipyretic, and hemostatic effects. *Rt* contains twenty compounds including anthraquinones that possess anti-inflammatory effects [9]. However, little information is available about anti-inflammatory and antioxidative activities of *Rt*.

Interleukin-10 (IL-10) is known to inhibit the lipopolysaccharide- (LPS-) induced production of several inflammatory mediators, the expression of cytokine receptors, and the expression of major histocompatibility complex II in microglia [10–15]. IL-10 can also inhibit the LPS- or cytokine-induced expression of chemokines and adhesion molecules in microglia [16, 17]. Furthermore, a peripheral injection with LPS in IL-10-deficient mice causes a prominent cognitive deficit compared with wild-type mice [18]. IL-10 acts through the activation of its receptor (IL-10R). Upon binding, the receptor oligomerizes into a tetramer composed of two ligand-binding subunits (IL-10R1) and two accessory subunits (IL-10R2) that in turn activate an intracellular signaling cascade [19]. Therefore, IL-10 is considered a potent negative autocrine regulator of microglia, as microglia produce IL-10 and possess IL-10R [11, 20]. Resveratrol, a natural polyphenol first identified as a bioactive compound in 1992, is naturally present in red wine and grapes and has been to exert a neuroprotective effect through its anti-inflammatory and antioxidant effects [21]. Recently, the production and secretion of IL-10 from microglia has shown to be responsible for the anti-inflammatory and antioxidant effects of this agent [22, 23]. Moreover, it has been also reported that the protective effects of curcumin are IL-10 dependent [24].

In the present study, we thus examined the possible antineuroinflammatory and antioxidative effects of *Rt* using the organotypic hippocampal slice cultures and cultured microglia after stimulation with endogenous microglial activators localized in the senile plaques of AD patients.

## 2. Materials and Methods

### 2.1. Reagents

*Rt* was purchased from Qinghai Jinke Tibetan Medicine Pharmaceutical Co., Ltd. (Xining, China). *Rt* contained seven anthraquinones or glycosides of anthraquinones including chrysophanol, aloe-emodin, physcion, rhein, emodin, chrysophanol-8-O-*β*-D-glucopyranoside, and aloe-emodin-8-O-*β*-D-glucopyranoside. In addition, *Rt* contained two phenylbutanone glucopyranosides (lindleyin and isolindleyin), piceatannol, (+)-catechin, *β*-sitosterol, epicatechin-3-O-gallate, and torachrysone-8-O-*β*-D-glucopyranoside. The suitable concentration of methanol for cell culture was titrated in order to prevent the interference induced by the methanol solvent. Chromogranin A (CGA, synthetic human CGA286-301) and pancreastatin (PST) were purchased from Peptide Institute (Osaka, Japan). Mouse anti-IL-10 neutralizing antibody (IL-10NAb) was purchased from Abcam (Cambridge, UK). Antibodies against mouse anti-interleukin-1*β* (IL-1*β*), mouse antiphospho-I*κ*B*α*, rabbit anti-I*κ*B*α*, and goat antiphospho-signal transducer and activator of transcription 1 (STAT1) were purchased from Santa Cruz Biotechnology (Santa Cruz, CA, USA). Major *Rt* components including chrisophanol, physcion, *β*-sitosterol, emodin, aloe-emodin, (+)-catechin, and piceatannol were purchased from Abcam (UK). In preliminary experiments, minimum effective dose of either CGA or PST for mRNA expression of proinflammatory mediators in MG6 cells and primary microglia was determined to be 10 nM. Therefore, CGA and PST with the concentration of 10 nM were used in this study. On the other hand, three different concentrations (10, 30, and 100 *μ*M) of aloe-emodin, (+)-catechin, piceatannol, chrisophanol, physcion, *β*-sitosterol, and emodin were used.

### 2.2. Cell Viability Assay

MG6 cells were seeded in 96-well plates (5 × 10^3^ cells/well) overnight [25]. Different concentrations ranging from 5 to 500 *μ*g/ml of *Rt* were treated for 24 h. A cell viability assay was conducted using a cell counting kit (Dojindo, Japan) according to the protocol provided by the manufacturer. The optical density was read at wavelength of 450 nm with a microplate reader. The cell viability was calculated by dividing the optical density of *Rt*-treated group by that of nontreated group. *Rt* up to 10 *μ*g/ml had no significant toxic effect on MG6 cells, therefore 10 *μ*g/ml *Rt* was used in further experiments.

### 2.3. Organotypic Hippocampal Slice Cultures

Male C57BL/6 mice (10 months of age) were sacrificed and their brains were removed. All animals were treated in accordance with the protocols approved by the animal care and use committee of Kyushu University. Sagittal sections 200 *μ*m thick were cut using a vibratome (VT1000S; Wetzlar, Leica), and intact sections were carefully selected under a microscope and incubated in cooled dissection buffer (50% HEPES-buffered MEM, 1% penicillin-streptomycin, 10 mM Tris, pH 7.2) for 30 min at 4°C. The slices were then carefully transferred to 24-well plates containing 0.5 ml of slice culture medium (50% HEPES-buffered MEM, 25% heat-inactivated horse serum, 25% HBSS, 1 mM L-glutamine, pH; 7.4) and maintained in a cell culture incubator at 37°C, 5% CO_2_. One day after preparation, the medium was changed, and *Rt* was applied at 10 *μ*g/ml. CGA (10 nM) and PST (10 nM) were added 24 h after *Rt* treatment. The slices were collected and lysed for Western blotting at 48 h after treatment with CGA or PST. In some experiments, microglia were depleted from hippocampal slice cultures using saponin coupled to an antibody against Mac1 (Mac1-sap; Advanced Targeting Systems, San Diego, USA). Mac1-sap at 1.3 nM was applied to hippocampal slice cultures 24 h prior to stimulation with CGA or PST.

### 2.4. Microglia Cell Culture

The c*-myc*-immortalized mouse microglial cell line MG6 (Riken Cell Bank, Tsukuba, Japan) was maintained in DMEM supplemented with 100 *μ*mol/L *β*-mercaptoethanol, 10 *μ*g/mL of insulin, 1% penicillin-streptomycin (Invitrogen, Grand Island, NY, USA), 4500 mg/L glucose (Invitrogen), and 10% FBS according to previously described methods. Primary cultured microglia were isolated from the mixed primary cell cultures obtained from the cerebral cortex of three-day-old C57BL/6 mice according to previously described methods [26].

### 2.5. Real-Time Quantitative Polymerase Chain Reaction (qRT-PCR)

Total RNA was extracted using RNAiso Plus according to the manufacturer's instructions. 1 *μ*g of total RNA was used for cDNA synthesis using the QuantiTect Reverse Transcription Kit (Qiagen, Hilden, Germany). After an initial amplification with a denaturation step at 95°C for 5 m, followed by 30–40 cycles of denaturation at 95°C for 5 s, annealing at 60°C for 10 s, and extension at 72°C for 30 s, a final extension at 72°C for 5 m was done upon completion of the cycling steps. The cDNA was amplified in duplicate using a Rotor-Gene SYBR Green RT-PCR Kit (Qiagen) with a Corbett Rotor-Gene RG-3000A Real-Time PCR System. The data were evaluated using the RG-3000A software program (version Rotor-Gene 6.1.93, Corbett, Sydney, Australia). The sequences of primer pairs were described as follows: IL-1*β*: 5′-CAACCAACAAGTGATATTCTCCATG-3′ and 5′-GATCCACACTCTCAGCTGCA-3′; inducible nitric oxide synthase (iNOS): 5′-GCCACCAACAATGGCAAC-3′ and 5′- CGTACCGGATGAGCTGTGAATT- 3′; TNF-*α*: 5′-ATGGCCTCCCTC TCAGTTC -3′ and 5′-TTGGTGGTTTGCTACGACGTG-3′; and IL-10: 5′-GACCAGCTGGACAACATACTGC TAA-3′ and 5′-GATAAGGATTGGCAACCCAAGTAA-3′. For data normalization, an endogenous control (actin) was assessed to control for the cDNA input, and the relative units were calculated by a comparative Ct method. All qRT-PCR experiments were repeated three times, and the results are presented as the means of the ratios ± SEM.

### 2.6. Western Blotting

MG6 were cultured at a density of 5 × 10^5^ cells/mL. After treatment with *Rt* (10 *μ*g/ml) for 24 h, microglia were treated with CGA (10 nmol/L) for various time points. The cytosolic samples were collected at various time points. Western blotting was performed with a SDS-PAGE electrophoresis system. 30 *μ*g protein samples were resuspended in sample buffer, then electrophoresed on a 15% or 12% Tris gel, and then blotted to the PVDF membrane. After blocking, the membranes were incubated at 4°C overnight under gentle agitation with each primary antibody: mouse anti-IL-1*β* (1 : 1000), mouse antiphospho-I*κ*B*α* (1 : 1000), rabbit anti-I*κ*B*α* (1 : 1000), goat antiphospho-STAT1 (1 : 1000), and anti-*β*-actin (1 : 1000) antibodies overnight at 4°C. After washing, the membranes were incubated with horseradish peroxidase- (HRP-) labeled antigoat (1 : 1000; R&D Systems, Minneapolis, MN, USA), antirabbit (1 : 1000; Beckman Coulter, Tokyo, Japan), or antimouse (1 : 1000; Amersham Pharmacia Biotech, Piscataway, NJ, USA) antibodies for 2 h at 24°C and then detected using an enhanced chemiluminescence detection system (ECK kit; Amersham Pharmacia Biotech, Piscataway, NJ, USA) with an image analyzer (LAS-4000; Fuji Photo Film, Tokyo, Japan).

### 2.7. ELISA

The cytokines IL-1*β* and IL-10 were measured by enzyme-linked immunosorbent assay (ELISA) kits (R&D Systems) following the protocol provided by the manufacturer. The absorbance at 450 nm was determined using a microplate reader.

### 2.8. Immunostaining

The cultured microglia were fixed with 4% paraformaldehyde 48 h after CGA treatment or pretreatment with *Rt*. They were then incubated with the mouse anti-p65 overnight at 4°C. After washing with PBS, the sections were incubated with donkey antimouse Alexus 488 (1 : 500; Jackson ImmunoResearch, West Grove, PA, USA), then incubated with Hoechst (1 : 200), and mounted in Vectashield antifading medium (Vector Laboratories, Burlingame, CA, USA). Fluorescence images were taken using a confocal laser-scanning microscope (CLSM; 2si Confocal Laser Microscope, Nikon, Tokyo, Japan). The line plot profile was analyzed using Image J.

### 2.9. NO_2_^−^/NO_3_^−^ Assay

MG6 were cultured at a density of 5 × 10^5^ cells/mL. After treatment with *Rt* (10 *μ*g/L) for 24 h, microglia were treated with CGA (10 nM) for 72 h, and the supernatant of the cells was collected. The amounts of NO_2_^−^ and NO_3_^−^ were measured by NO_2_^−^/NO_3_^−^ assay kits (R&D Systems) following the protocol provided by the manufacturer. The absorbance at 540 nm was determined using a microplate reader.

### 2.10. Statistical Analyses

The data are represented as the mean ± standard error of the mean.

A two-tailed unpaired Student's *t*-test and a one-way analysis of variance (ANOVA) with a post hoc Tukey's test were performed for the statistical analyses by the GraphPad Prism 7 Software package (GraphPad Software Inc., San Diego, CA, USA). A value of *P* < 0.05 was considered to indicate statistical significance.

## 3. Results

### 3.1. Inhibitory Effects of *Rt* on the CGA- and PST-Induced Expression of IL-*β* in Organotypic Hippocampal Slice Cultures

**Figure 1:**
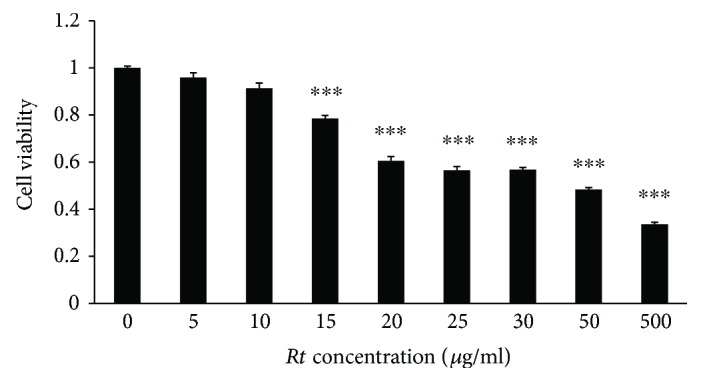
The cell viability of MG6 cells at 24 h after treatment with different dose of *Rt* by using a cell counting kit-8. The results represent the mean ± SEM of four independent experiments. The asterisks indicate a statistically significant difference from the value in untreated cells (^∗∗∗^*P* < 0.001).

The viability of MG6 cells was examined using the CCk-8 assay at 24 h after treatment with *Rt* with the concentration ranging from 5 to 500 *μ*g/ml. *Rt* up to 10 *μ*g/ml had no significant toxic effect on MG6 cells (Figure 1). CGA and PST with the minimum effective dose determined in preliminary experiments were used to activate microglia, as they are potent endogenous microglial activators and localize in the senile plaques of AD patients [27–29].

**Figure 2:**
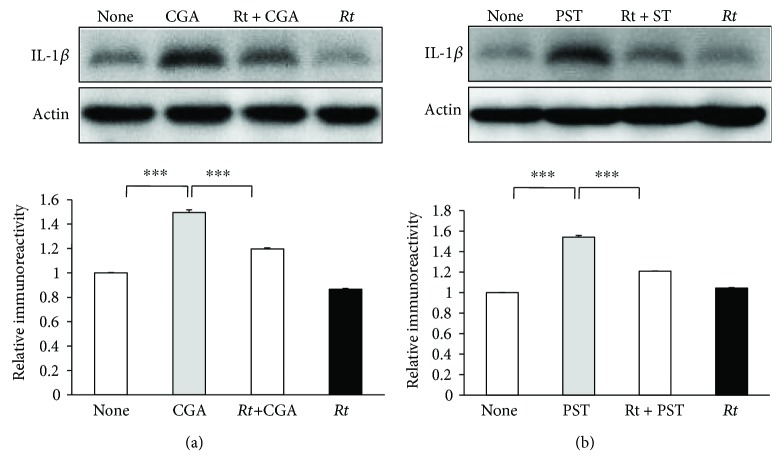
Inhibitory effects of *Rt* on CGA- or PST-induced IL-1*β* production in the hippocampal slice cultures. (a) The effect of *Rt* (10 *μ*g/ml) on the protein level of IL-1*β* after stimulation with CGA using Western blotting. The results represent the mean ± SEM of three independent experiments. The asterisks indicate a statistically significant difference from the indicated value (^∗∗∗^*P* < 0.001). (b) The effect of *Rt* (10 *μ*g/ml) on the protein level of IL-1*β* after stimulation with PST. The results represent the mean ± SEM of three independent experiments. The asterisks indicate a statistically significant difference from the indicated value (^∗∗∗^*P* < 0.001).

**Figure 3:**
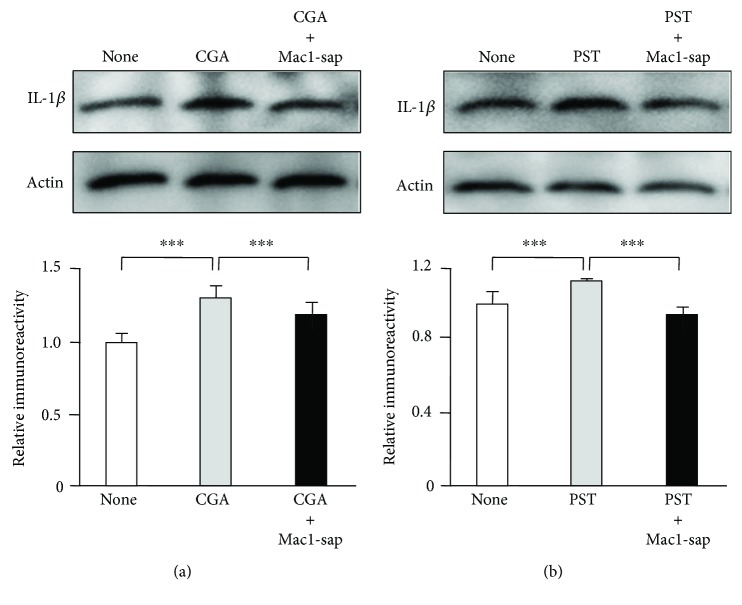
The failure of IL-1*β* production after treatment with CGA or PST in the microglia-depleted hippocampal slice cultures using Mac1-sap. (a) The effect of CGA on the protein level of IL-1*β* in the nontreated and Mac1-sap-treated cultures. The results represent the mean ± SEM of three independent experiments. The asterisks indicate a statistically significant difference from the indicated value (^∗∗∗^*P* < 0.001). (b) The effect of PST on the protein level of IL-1*β* in the nontreated and Mac1-sap-treated culture. The results represent the mean ± SEM of three independent experiments. The asterisks indicate a statistically significant difference from the indicated value (^∗∗∗^*P* < 0.001).

To elucidate the possible antineuroinflammatory effects, the effects of *Rt* (10 *μ*g/ml) on the expression of IL-*β* in organotypic hippocampal slice cultures were examined by Western blotting. The mean protein level of IL-1*β* was significantly increased in the organotypic hippocampal slice cultures at 48 h after stimulation with CGA (10 nM). *Rt* significantly suppressed the mean protein level of IL-1*β* in CGA-stimulated organotypic hippocampal slice cultures (Figure 2(a)). PST (10 nM) also significantly increased the mean protein level of IL-*β* in the organotypic hippocampal slice cultures to a similar extent as CGA. Furthermore, *Rt* significantly suppressed the PST-induced IL-1*β* production (Figure 2(b)). Therefore, CGA286-301 may be an active component of PST, as CGA used in this study was human CGA286-301, which includes the carboxy-terminal of PST. In contrast, both CGA and PST failed to significantly increase the mean protein level of IL-1*β* in the Mac1-sap treated hippocampal slice cultures (Figures 3(a) and 3(b)), suggesting that microglia are responsible for the IL-1*β* production after treatment with CGA or PST.

### 3.2. Inhibitory Effects of *Rt* on the CGA-Induced Expression of Proinflammatory and Oxidative Mediators in Microglia

**Figure 4:**
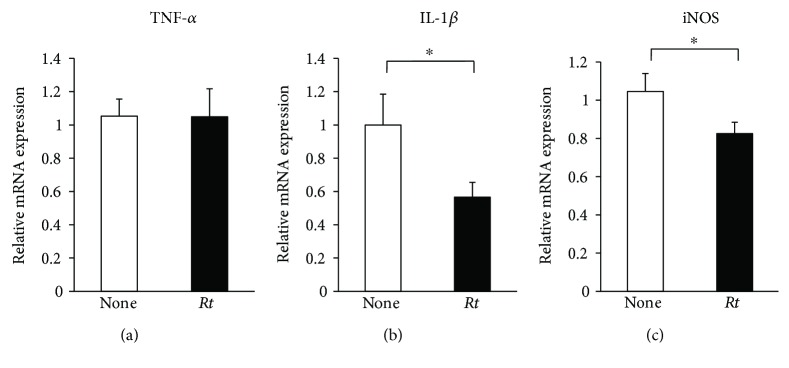
The inhibitory effects of *Rt* on the CGA-induced expression of proinflammatory and oxidative mediators in microglia. The effect of *Rt* (10 *μ*g/ml) on the basal mRNA expression of TNF-*α* (a), IL-1*β* (b), and iNOS (c) in the nonstimulated MG6 cells. Each column and bar represents the mean ± SEM (*n* = 4 each). The asterisks indicate a statistically significant difference from the indicated value (^∗^*P* < 0.05).

**Figure 5:**
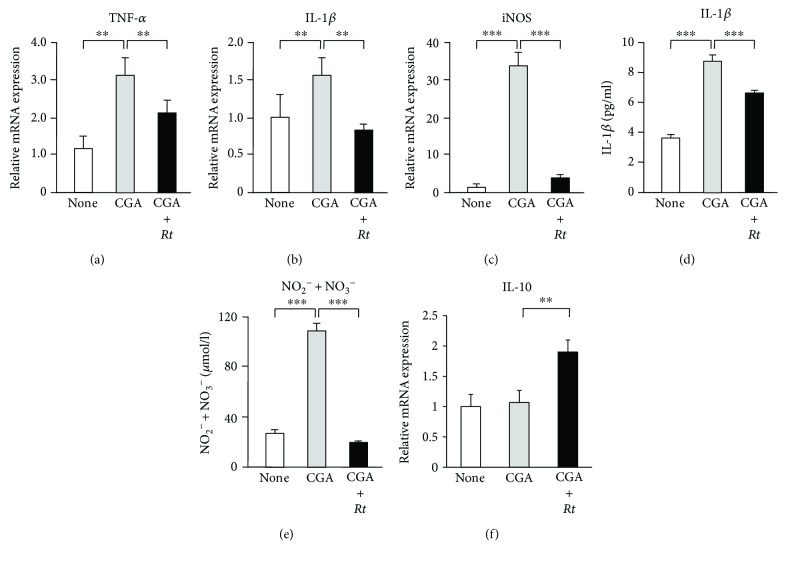
The inhibitory effects of *Rt* on the CGA-induced expression of proinflammatory and oxidative mediators in microglia. (a–c) The effect of *Rt* (10 *μ*g/ml) on the mRNA expression of TNF-*α* (a), IL-1*β* (b), and iNOS (c) in the MG6 cells after stimulation with CGA. Each column and bar represents the mean ± SEM (*n* = 4 each). The asterisks indicate a statistically significant difference from the indicated value (^∗∗^*P* < 0.01, ^∗∗∗^*P* < 0.001). (d) The secretion of IL-1*β* in the MG6 cells 96 h after stimulation with CGA with or without pretreatment with *Rt*. The results represent the mean ± SEM of three independent experiments. The asterisks indicate a statistically significant difference from the indicated value (^∗∗∗^*P* < 0.001). (e) The examination of NO metabolites in the culture medium of MG6 cells 72 h after stimulation with CGA with or without pretreatment with *Rt* by NO_2_^−^/NO_3_^−^ assay. The results represent the mean ± SEM of three independent experiments. The asterisks indicate a statistically significant difference from the indicated value. (^∗∗∗^*P* < 0.001). (f) The effect of *Rt* (10 *μ*g/ml) on the mRNA expression of IL-10 in the MG6 cells after stimulation with CGA. The results represent the mean ± SEM of four independent experiments. The asterisks indicate a statistically significant difference from the indicated value (^∗∗^*P* < 0.01).

To elucidate the possible anti-inflammatory and antioxidative roles, effects of *Rt* (10 *μ*g/ml) on the expression of proinflammatory mediators by microglia were examined at the transcriptional level using quantitative RT-PCR. *Rt* significantly suppressed the mean basal mRNA expression levels of IL-1*β* and iNOS, but not TNF-*α*, in nonstimulated MG6 cells (Figures 4(a)–4(c)). The mean mRNA expression levels of TNF-*α*, IL-1*β*, and iNOS was significantly increased in MG6 cells at 24 h after stimulation with CGA (10 nM). *Rt* significantly suppressed the mean mRNA expression levels of TNF-*α*, IL-1*β*, and iNOS in CGA-stimulated MG6 cells (Figures 5(a)–5(c)). Furthermore, the secretion of IL-1*β* and NO metabolites in the culture medium of MG6 cells was assessed by an ELISA and NO_2_^−^/NO_3_^−^ assay, respectively. The mean levels of IL-1*β* and NO_2_^−^/NO_3_^−^ were significantly increased in the culture medium of MG6 cells at 24 h after treatment with CGA. *Rt* also significantly decreased the mean levels of IL-1*β* and NO_2_^−^/NO_3_^−^ in the culture medium of MG6 cells (Figures 5(d) and 5(e)).

**Figure 6:**
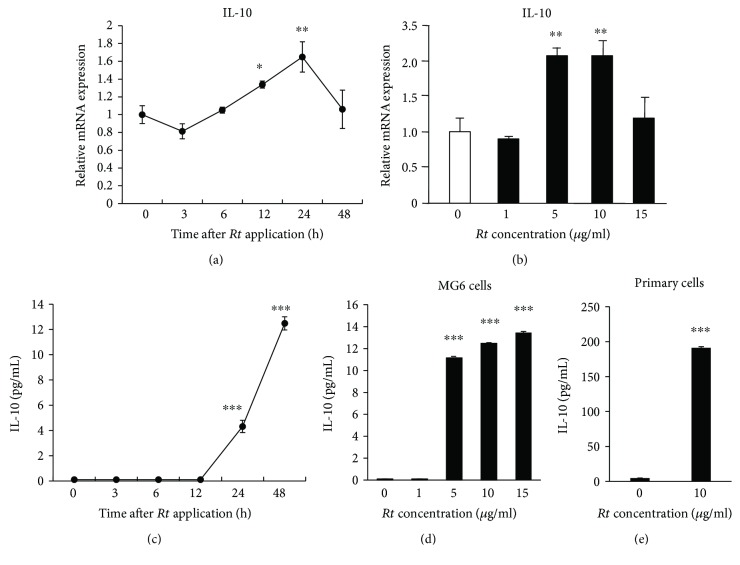
The capable regulation of IL-10 by *Rt* in MG6 cells and primary cultured microglia. (a) The mRNA changes of IL-10 at different time points after treatment with *Rt* (10 *μ*g/ml). The results represent the mean ± SEM of three independent experiments. The asterisks indicate a statistically significant difference from the value at the start of experiments (0 h) (^∗^*P* < 0.05, ^∗∗^*P* < 0.01). (b) The mRNA changes of IL-10 24 h after treatment with different doses of *Rt*. The results represent the mean ± SEM of three independent experiments. The asterisks indicate a statistically significant difference from the value in untreated cells (^∗∗^*P* < 0.01). (c) The secretion of IL-10 in the cultured medium of MG6 cells at different time points after treatment with *Rt* (10 *μ*g/ml). The results represent the mean ± SEM of three independent experiments. The asterisks indicate a statistically significant difference from the value at the start of experiments (0 h) (^∗∗∗^*P* < 0.001). (d) The secretion of IL-10 in the cultured medium of MG6 cells 24 h after treatment with different doses of *Rt*. The results represent the mean ± SEM of three independent experiments. The asterisks indicate a statistically significant difference from the value in untreated cells (^∗∗∗^*P* < 0.001). (e) The secretion of IL-10 in the cultured medium of primary cultured microglia 24 h after treatment with *Rt* (10 *μ*g/ml). The results represent the mean ± SEM of three independent experiments. The asterisks indicate a statistically significant difference from the value in untreated cells (^∗∗∗^*P* < 0.001).

RT-PCR and ELISA were also performed to determine whether or not *Rt* was able to regulate the production of the anti-inflammatory cytokine IL-10. CGA (10 nM) alone failed to increase the mean mRNA level of IL-10, whereas the combination of CGA and *Rt* significantly increased the mean mRNA level of IL-10 (Figure 5(f)), suggesting that *Rt* was able to upregulate the expression of IL-10. As expected, the mean mRNA expression of IL-10 in MG6 cells was significantly increased at 12 h and peaked at 24 h after treatment with *Rt* alone at 5 *μ*g/ml (Figure 6(a)). *Rt* was able to significantly increase the mean mRNA expression level of IL-10 in MG6 cells at 24 h after treatment at >5 *μ*g/ml (Figure 6(b)) without affecting the expression of IL-1*β* (data not shown). In contrast, the mean level of IL-10 in the culture medium of MG6 cells significantly increased at 24 h and peaked at 48 h after treatment of *Rt* at 5 *μ*g/ml (Figure 6(c)). The mean level of IL-10 secretion was significantly increased at 48 h after treatment with *Rt* at >5 *μ*g/ml in the culture medium of both MG6 cells and primary cultured murine microglia (Figure 6(d)). Of note, the mean level of IL-10 in the culture medium of primary cultured microglia (200 pg/mL) was more than tenfold greater than that of MG6 cells (14 pg/mL) after treatment with *Rt* (10 *μ*g/mL).

### 3.3. Possible Role of IL-10 in the Anti-Inflammatory Effects of *Rt* on CGA-Stimulated Microglia

**Figure 7:**
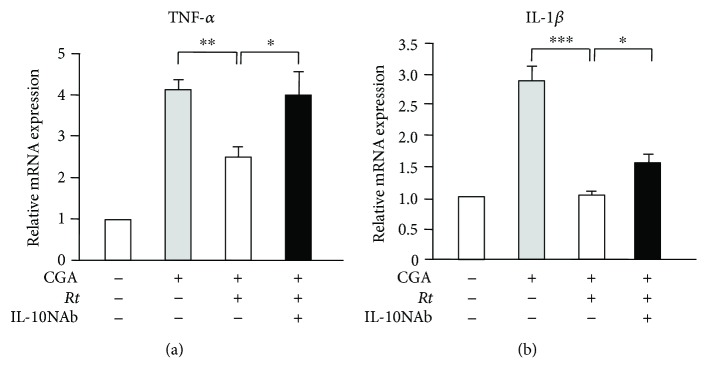
Possible role of IL-10 in the anti-inflammatory effects of *Rt* on CGA-stimulated microglia. (a, b) The mRNA changes of TNF-*α* (a) and IL-1*β* (b) of CGA stimulated MG6 cells in the presence or absence of *Rt* (10 *μ*g/ml) and IL-10 neutralizing antibody (IL-10NAb). The results represent the mean ± SEM of three independent experiments. The asterisks indicate a statistically significant difference from the indicated value (^∗^*P* < 0.05, ^∗∗^*P* < 0.01, ^∗∗∗^*P* < 0.001).

To investigate the involvement of IL-10 in the anti-inflammatory effects of *Rt* (10 *μ*g/ml) on inflammatory responses of microglia, we evaluated the effects of IL-10NAb on the *Rt*-mediated inhibition of the TNF-*α* and IL-1*β* expression in CGA-stimulated MG6 cells. IL-10NAb inhibited the mean mRNA expression of TNF-*α* and IL-1*β* in CGA-stimulated MG6 cells in the presence of *Rt* (Figures 7(a) and 7(b)). There was no significant difference between the mean percentage recovery from *Rt*-induced inhibition of TNF-*α* and IL-1*β* mRNA expression after treatment with IL-10NAb (66.7% for TNF-*α* and IL-1*β* for 63.6%). These observations strongly suggest that IL-10 plays a critical role in the anti-inflammatory property of *Rt* in microglia.

### 3.4. Effects of *Rt* on the Nuclear Factor (NF)-*κ*B and STAT1 Activation Pathways in CGA-Stimulated Microglia

**Figure 8:**
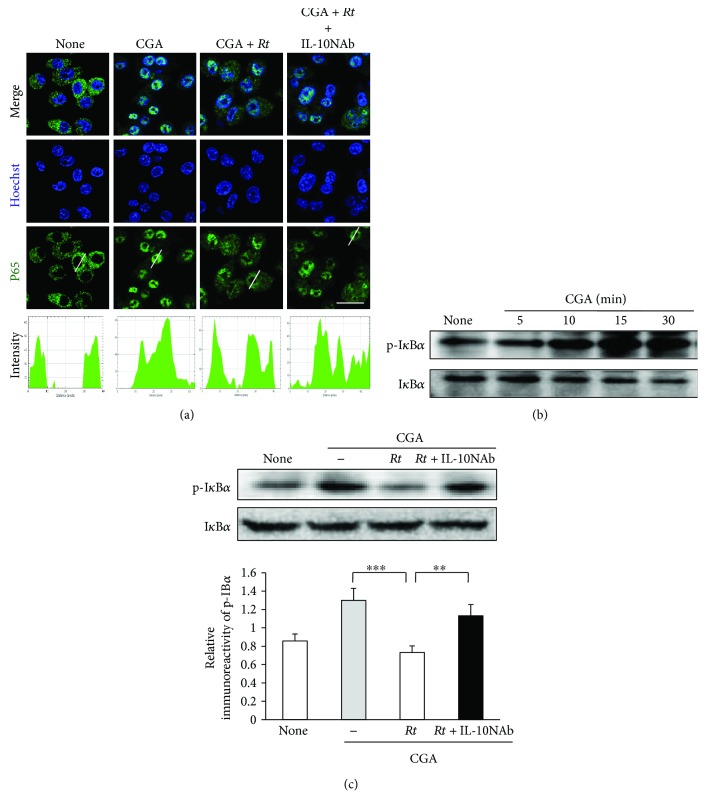
Effects of *Rt* on the NF-*κ*B activation pathways in CGA-stimulated microglia. (a) Immunofluorescence CLMS images indicating the nuclear translocation of p65 (green) in MG6 cells with Hoechst-stained nuclei (blue) at 24 h after stimulation with CGA (10 nM) in the presence or absence of *Rt* (10 *μ*g/ml) and IL-10NAb. Scale bar, 15 *μ*m. The typical cells were analyzed by line plot profile to show the cytosol and nuclear location of p65. (b) The protein expression of phospho-I*κ*B*α* at different time points after stimulation with CGA. (c) The protein expression of phospho-I*κ*B*α* in MG6 cells 30 min after CGA stimulation in the presence or absence of *Rt* (10 *μ*g/ml) and IL-10NAb and the quantitative analyses of the immunoblotting for phospho-I*κ*B*α*. The results represent the mean ± SEM of four independent experiments. The asterisks indicate a statistically significant difference from the indicated value (^∗∗^*P* < 0.01, ^∗∗∗^*P* < 0.001).

The effects of *Rt* on the nuclear translocation of p65 induced by treatment with CGA were examined. *Rt* (10 *μ*g/ml) significantly inhibited the CGA-induced nuclear translocation of p65 in MG6 cells (Figure 8(a)). When IL-10 was neutralized, the CGA-induced nuclear translocation of p65 in MG6 cells returned to levels in the absence of *Rt* (Figure 8(a)). These observations clearly show that *Rt* can inhibit CGA-induced activation of the NF-*κ*B pathway in MG6 cells through production of IL-10.

**Figure 9:**
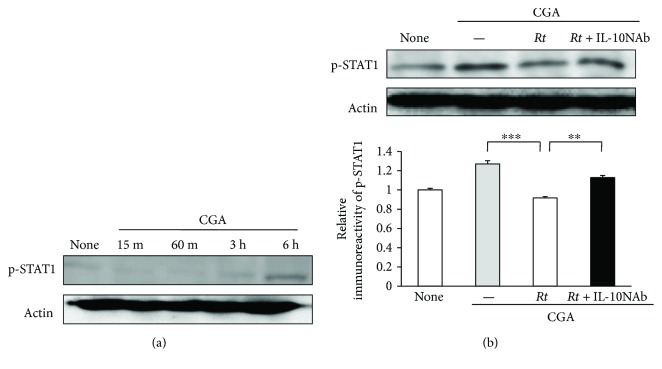
Effects of *Rt* on the STAT1 activation pathways in CGA-stimulated microglia. (a) The protein expression of phospho-STAT1 at different time points after stimulation with CGA. (b) The protein expression of phospho-STAT1 in MG6 cells 6 h after CGA stimulation in the presence or absence of *Rt* (10 *μ*g/ml) and IL-10NAb and the quantitative analyses of the immunoblotting for phospho-STAT1. The results represent the mean ± SEM of four independent experiments. The asterisks indicate a statistically significant difference from the indicated value (^∗∗^*P* < 0.01, ^∗∗∗^*P* < 0.001).

The activation of NF-*κ*B and STAT1 pathways were further examined after treatment with CGA (10 nM) in MG6 cells by immunoblotting, as these two signaling pathways are required for the production of TNF-*α*, IL-1*β*, and NO. The mean levels of phospho-I*κ*B*α* and phospho-STAT1 were significantly increased in MG6 cells after treatment with CGA at 10 min and 3 h, respectively (Figures 8(b) and 9(a)). *Rt* at 10 *μ*g/ml significantly decreased the mean levels of phospho-I*κ*B*α* (Figure 8(c)) and phospho-STAT1 (Figure 9(b)) in CGA-stimulated MG6 cells. Furthermore, the neutralization of IL-10 using IL-10NAb significantly inhibited the mean levels of phospho-I*κ*B*α* (Figure 8(c)) and phospho-STAT1 (Figure 9(b)) in CGA-stimulated MG6 cells in the presence of *Rt*. These observations suggest that IL-10 plays a critical role in the *Rt*-induced anti-inflammatory antioxidative effects through the suppression of NF-*κ*B and STAT1 activation pathways.

### 3.5. Possible Components of *Rt* Responsible for the Production of IL-10 by Microglia

**Figure 10:**
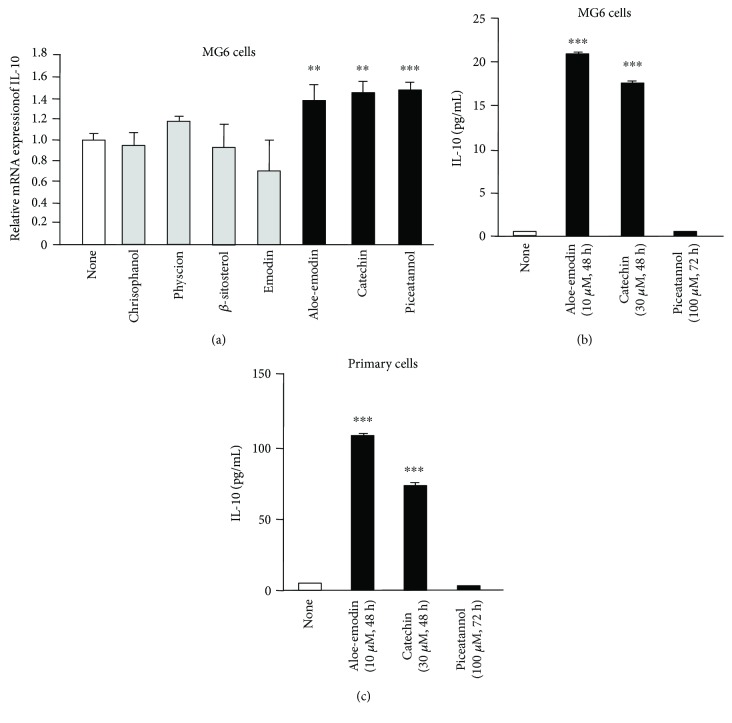
Possible components of *Rt* responsible for the production of IL-10 in microglia. (a) The change in the mRNA of IL-10 in MG6 cells 24 h after treatment with components of *Rt*, *including* chrisophanol, physcion, *β*-sitosterol, emodin, aloe-emodin, (+)-catechin, and piceatannol. The results show the relative mRNA expression of IL-10 after treatment with aloe-emodin (10 *μ*M), (+)-catechin (30 *μ*M), and piceatannol (100 *μ*M) with the minimum effective doses, which were determined after treatment with three different doses (10, 30, and 100 *μ*M) for 24 h. On the other hand, chrisophanol, physcion, *β*-sitosterol, and emodin with the concentration of 100 *μ*M were applied for 24 h. The results represent the mean ± SEM of three independent experiments. The asterisks indicate a statistically significant difference from the indicated value (^∗∗^*P* < 0.01, ^∗∗∗^*P* < 0.001). (b) The secretion of IL-10 in the medium of MG6 cells and primary cultured microglia after treatment with aloe-emodin, (+)-catechin, and piceatannol. The results show the mean IL-10 secreted in the culture medium after treatment with aloe-emodin (10 *μ*M for 48 h) and (+)-catechin (30 *μ*M for 48 h) with the minimum effective doses for the most effective experimental time points. The most effective experimental time points were determined after examination of three different experimental time points (24, 48 and 72 h). On the other hand, piceatannol with 100 *μ*M was applied for 72 h. The results represent the mean ± SEM of three independent experiments. The asterisks indicate a statistically significant difference from the value in untreated cells (^∗∗∗^*P* < 0.001).

Finally, to explore the components of *Rt* responsible for the production of IL-10 in microglia, RT-PCR was performed to examine the effects of major components of *Rt* including on the mRNA expression in MG6 cells. Effects of three different concentrations (10, 30, and 100 *μ*M) of major *Rt* components including chrisophanol, physcion, *β*-sitosterol, emodin, aloe-emodin, (+)-catechin, and piceatannol were examined at 24 h on the mRNA expression of IL-10 in MG6 cells. Among the major components of *Rt*, three components, namely, aloe-emodin, (+)-catechin, and piceatannol, were found to significantly increase the mRNA expression of IL-10 in MG6 cells at 24 h after treatment (Figure 10(a)). The minimum effective doses of aloe-emodin, (+)-catechin, and piceatannol were determined to be 10, 30, and 100 *μ*M, respectively. In contrast, chrisophanol, physcion, *β*-sitosterol, or emodin at concentrations up to 100 *μ*M had no effect on the mRNA expression of IL-10 in MG6 cells.

Then, effects of aloe-emodin, (+)-catechin, and piceatannol with the most effective doses on the secretion of IL-10 from MG6 cells and primary cultured microglia were examined at three different experimental time points (24, 48, and 72 h).  Figure 10(b) showed the mean amounts of IL-10 secreted in the culture medium of MG6 cells and primary cultured microglia after treatment of aloe-emodin and (+)-catechin with the minimum effective doses at the most effective experimental time points. On the other hand, piceatannol (100 *μ*M) failed to secrete IL-10 even at 72 h after treatment. Of note, the mean level of IL-10 in the culture medium of primary cultured microglia was approximately tenfold greater than that of MG6 cells.

## 4. Discussion

In the present study, we demonstrated the antineuroinflammatory effects of *Rt* in CGA-stimulated organotypic hippocampal slice cultures, MG6 cells, and primary cultured microglia. *Rt* with the concentration of 10 *μ*g/mL significantly suppressed the CGA-induced production of IL-1*β* in the organotypic hippocampal cultures. *Rt* at >5 *μ*g/mL also significantly suppressed the CGA-induced expression of TNF-*α*, IL-1*β*, and NO, major molecules produced by neurotoxic microglia. Furthermore, we demonstrated that *Rt* alone was able to upregulate IL-10 at both the mRNA and protein levels, without the upregulation of proinflammatory and oxidative molecules. When IL-10 was neutralized, the mean expression levels of TNF-*α* and IL-1*β* returned to the levels observed in the absence of *Rt*. These observations indicate that *Rt* may act as a neuroprotective agent during neuroinflammation through inducing the production and secretion of IL-10, a potent negative autocrine regulator of microglia. More recently, IL-10 has been reported to alter macrophage function by promoting the clearance of damaged mitochondria and modulating cellular metabolism to inhibit inflammation [30]. Therefore, *Rt* may increase the capacity of microglia to produce IL-10 through enhanced clearance of damaged mitochondria. In this regard, *Rt* may act as a polarizing agent in microglia, favoring the shifting towards M2-like phenotype, more efficient as IL-10 producer.

The activation of the NF-*κ*B and STAT1 signaling pathways is considered to play a critical role in the polarization of microglia in the neurotoxic phenotype, as NF-*κ*B and STAT1 are transcription factors required for the production of TNF-*α*, IL-1*β*, and NO. The *Rt*-induced inhibition of the NF-*κ*B and STAT1 signaling pathways activated in CGA-stimulated microglia was inhibited by neutralizing antibody against IL-10. These observations suggest that the *Rt*-induced production of IL-10 is responsible for the anti-inflammatory and antioxidative effects of *Rt* in activated microglia through the inhibition of the NF-*κ*B and STAT1 signaling pathways. Of further note, the mean level of IL-10 in the culture medium of primary cultured microglia (200 pg/mL) was approximately seventeenfold greater than that of MG6 cells (10–14 pg/mL). MG6 cells are a mouse microglial cell line immortalized by a replication-deficient retroviral vector containing the human c-myc gene. Therefore, the c-MYC expression in microglia may result in an increased production of proinflammatory mediators and decreased production of IL-10.

We next examined the effects of seven components of *Rt*, including aloe-emodin, (+)-catechin, natannol, chrisophanol, physcion, *β*-sitosterol, and emodin, on the increased mRNA expression of IL-10 in microglia. Among them, aloe-emodin and (+)-catechin were able to induce the secretion of IL-10 from microglia. On the other hand, there was a discrepancy between mRNA expression and secretion of IL-10 following treatment with piceatannol. After mRNA expression, the secretion of IL-10 could be affected by many steps including translation, posttranslational modification and secretion. The present study does not allow us to exclude any of these steps.

(+)-Catechin is a naturally occurring polyphenolic compound that has been shown to have anti-inflammatory, antioxidant, and free radical-scavenging properties in vitro. (+)-Catechin has been shown to decrease the production of the proinflammatory cytokines, including IL-1*β* and TNF-*α*, and to enhance the production of the anti-inflammatory cytokine IL-10. (+)-Catechin suppresses the production of proinflammatory mediators by mouse microglia BV-2 cells and mitigation of NF-*κ*B through intracellular signaling cascades, including Akt, extracellular-signal-regulated kinase, p38 mitogen-activated protein kinase, and AMP-activated protein kinase [31]. In contrast, aloe-emodin is a major anthraquinone in aloe plants that contains a polyphenolic structure. Aloe-emodin has been also shown to suppress the LPS-induced production of NO and prostaglandin E_2_ in mouse macrophage RAW 264.7 cells [32]. Therefore, the present study suggests that the anti-inflammatory and antioxidant activities of (+)-catechin and aloe-emodin are due their abilities to induce the production and secretion of IL-10.

It has been also reported that the gene delivery of IL-10-adeno-associated virus significantly reduces neuroinflammation, enhances neurogenesis, and improves the spatial cognitive dysfunction in a transgenic AD mouse model [33]. Furthermore, it has been suggested that the IL-10 gene polymorphisms process, which favours the development of AD, reinforces the link between inflammation and cognitive decline in elderly people [34]. IL-10 has been shown to reduce IL-1*β* production by preventing the excessive generation of reactive oxygen species from complex II in damaged mitochondria and limiting the inflammasome activation in macrophages [30], which are characteristics of aged microglia associated with cognitive dysfunction [35, 36]. These findings support the notion that IL-10 may ameliorate neuroinflammation, cognitive dysfunction, and neurodegeneration associated with AD. Therefore, *Rt* may be useful for the pharmacological intervention against excessive inflammatory and oxidative responses associated with AD by inducing the production of IL-10 by microglia, because some of active components of *Rt* including (+)-catechin can cross the blood-brain barrier [37].

## 5. Conclusions

*Rt* downregulates the production of proinflammatory and oxidative mediators, including IL-1*β*, TNF-*α*, and NO, by cultured activated microglia through the production of IL-10. Two components of *Rt*, aloe-emodin and (+)-catechin, are deemed responsible for the antineuroinflammatory and antioxidative effects of *Rt* through the secretion of IL-10 from microglia. Therefore, *Rt* may be useful for the pharmacological intervention against excessive inflammatory and oxidative responses associated with AD by inducing the production of IL-10 by microglia.
